# Effect of the insulin plant (*Costus igneus*) leaves on dexamethasone-induced hyperglycemia

**DOI:** 10.4103/0974-7788.64396

**Published:** 2010

**Authors:** Akhila J Shetty, Divya Choudhury, Rejeesh, Vinod Nair, Maria Kuruvilla, Shashidhar Kotian

**Affiliations:** *Department of Pharmacology, Kasturba Medical College (KMC), Mangalore Campus, Manipal University, Manipal, India*; 1 *Kasturba Medical College (KMC), Mangalore Campus, Manipal University, Manipal, India*; 2 *Department of Pharmacology, KSHEMA, Mangalore, India*; 3 *Department of Pharmacology, AIIMS, New Delhi, India*; 4 *Department of Forensic Medicine, AJIMS, New Delhi, India*; 5 *Department of Community Medicine, Kasturba Medical College (KMC), Mangalore Campus, Manipal University, Manipal, India*

**Keywords:** *Costus igeus*, hyperglycemia, insulin plant

## Abstract

*Costus igneus*, commonly known as insulin plant in India, belongs to the family Costaceae. Consumption of the leaves are believed to lower blood glucose levels, and diabetics who consumed the leaves of this plant did report a fall in their blood glucose levels. Objectives: The present study was planned to evaluate the effect of the leaves of *Costus igeus* on dexamethasone-induced hyperglycemia in male Wistar rats. Four groups of male Wistar rats (n= 6) were treated with 10 mg/kg/day of dexamethasone subcutaneously for 20 days. From day 11 to day 20, different groups received 100, 250 or 500 mg/kg/day of powdered leaves of *Costus igeus* in distilled water orally or Glibenclamide 500 µg/kg orally. On the 20th day, after overnight fasting, a retro-orbital puncture was performed for obtaining blood samples to estimate the fasting blood glucose level, and the same procedure was followed on the other eye 1 hour after a glucose load of 2.5 g/kg orally for estimation of post-glucose load blood glucose levels. Fasting blood sugar and postglucose load blood sugar levels were raised in the group that received dexamethasone when compared to normal controls (*P* < 0.001), whereas 250 and 500 mg/kg powdered leaf of *Costus igeus* and Glibenclamide 500 µg/kg decreased the dexamethasone-induced hyperglycemia (P < 0.01). The leaves of *Costus igeus* reduced the fasting and postprandial blood sugar levels, bringing them towards normal, in dexamethasone-induced hyperglycemia in rats.

## INTRODUCTION

**Figure 1:**
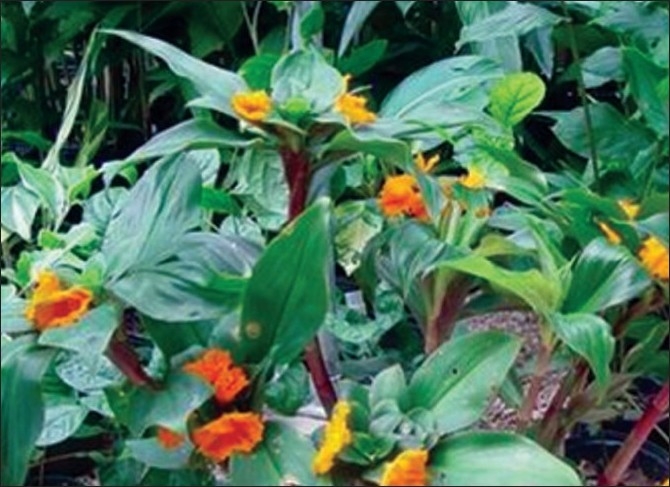
Photograph of the insulin plant (Costus igneus)

*Costus igeus*, [Figure 1] commonly known as insulin plant in India, belongs to family Costaceae. It is believed that consumption of the leaves helps lower the blood glucose levels, and diabetics who consumed the leaves of this plant report a fall in their blood glucose levels. This study was planned to evaluate the hypoglycemic effect of *Costus igeus*, on dexamethasone-induced hyperglycemia.

## MATERIALS AND METHODS

The study was conducted after obtaining approval from the Institutional Ethical Committee for Animal Experimentation. Male albino rats of Wistar strains were obtained from, and maintained in, the animal house, Department of Pharmacology, Kasturba Medical College, Mangalore, Karnataka, India. The rodents weighed 150-200 g and had access to food and water *ad libitum*. They were under natural light-and-dark cycles at a temperature of 28 ± 4°C, and were acclimatized for 3 days before the beginning of the experiment.

## Insulin plant (*Costus igeus*) leaf

The leaves were collected from the plants grown in Mangalore, Karnataka, India, over the months of September and October. Prior to use, the leaves were verified as belonging to the insulin plant (*Costus igeus*) by an Ayurvedic practitioner and dispenser, Dr. Bhandarkar, practicing in Mangalore, Karnataka, India. The leaves were shade-dried, finely powdered, weighed, ground in water using motor and pestle and used for the study.

## Glibenclamide

Commercially available Glibenclamide tablets were dissolved in water and administered orally to the rats at a dose of 500 µg/kg.[1]

Animals were divided into 6 groups. In each group, there were 6 rats. The present study was planned with 36 rats. Group 1 served as normal control; groups 2, 3, 4, 5, 6 received dexamethasone 10 mg/kg/day subcutaneously[2] for 10 days; on day 11, after overnight fasting, retro-orbital puncture was performed to obtain blood samples for estimation of fasting and postprandial blood sugar. Only those rats whose fasting and postprandial blood glucose levels were higher than those of the normal controls were utilized for further study. From day 11 to day 20, groups 2, 3, 4, 5, 6 continued to receive dexamethasone 10 mg/kg/day subcutaneously. Group 3 received 100 mg/kg/day of insulin plant leaf powder in 1 mL of distilled water per oral, in addition to dexamethasone. Group 4 received 250 mg/kg/day of insulin plant leaf powder in 1 mL of distilled water per oral, in addition to dexamethasone. Group 5 received 500 mg/kg/day of insulin plant leaf powder in 1 mL of distilled water per oral, in addition to dexamethasone. Group 6 received Glibenclamide 500 µg/kg per oral, in addition to dexamethasone.

On the 20^th^ day, after overnight fasting, retro-orbital puncture was done on the right eye to obtain blood for estimation of fasting blood glucose using glucometer. Immediately after this, a glucose load of 2.5 g/kg orally by gastric intubation was given and retro-orbital puncture was done on the other eye to measure the blood glucose level 1 hour after glucose load for estimation of postprandial blood glucose levels.

With dexamethasone, at different times, a few rats died, probably due to infection; as and when the rats died, we would include new rats and experiment with them from the beginning such that there were ultimately 6 rats per group. We had planned the present study with 36 rats, but we needed 45 rats for completion of the project.

## Statistical analysis

Kruskal-Wallis test (H) was applied for analysis of inter-group results. Mann-Whitney *U* test (Z) was applied for comparison of results between normal and dexamethasone-treated groups.

## RESULTS

**Figure 2:**
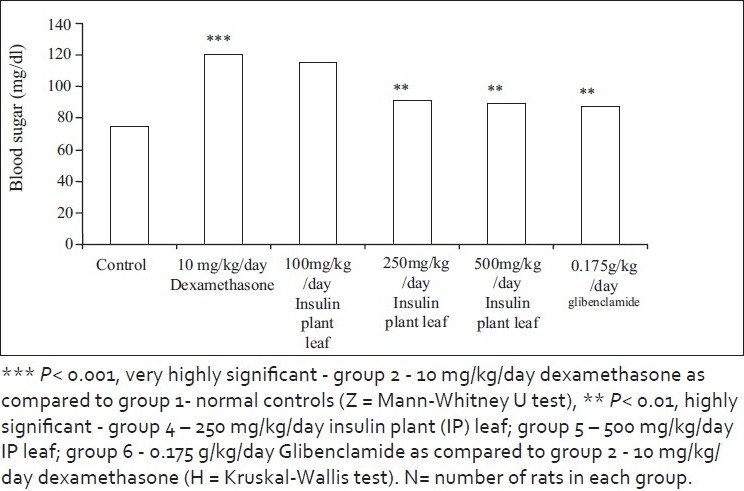
Effect on fasting blood sugar

**Figure 3:**
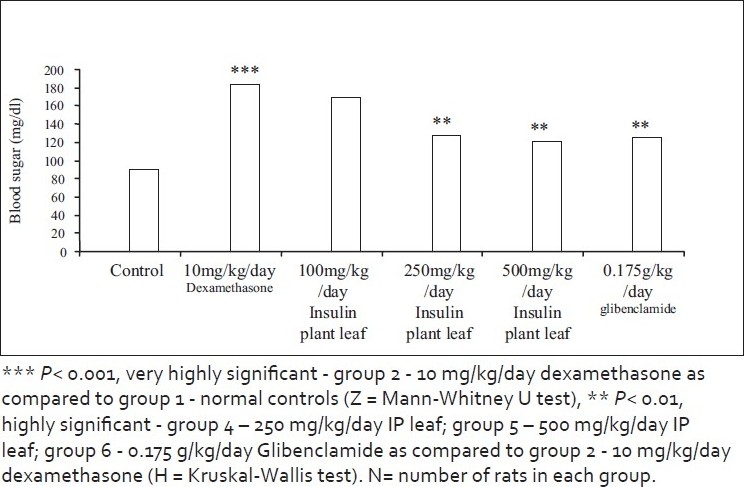
Effect on postprandial blood sugar

In the present study, there was increase in the fasting and postprandial glucose level with 10 mg/kg/day of dexamethasone for 10 days when compared to normal controls [*P* < 0.001, Mann-Whitney *U* test (Z)]. Reduction in the fasting and the postprandial blood sugar levels with leaves of insulin plant was comparable with that obtained with Glibenclamide 500 µg/kg at 250 mg/kg and 500 mg/kg of powdered leaves of the insulin plant (*Costus igeus*) [*P* < 0.01, Kruskal-Wallis test (H)] [Figures 2 and 3, Tables 1 and 2].

**Table 1:** Effect of insulin plant leaves and Glibenclamide on fasting blood glucose levels in dexamethasone-induced hyperglycemia

Groups	Mean	Std. deviation
Control	75.0	2.6
Dexamethasone (10 mg/kg/day)	120.3^***^	1.8
Insulin plant leaf (100 mg/kg/day)	115.0	1.7
Insulin plant (250 mg/kg/day)	91.0	1.4
Insulin plant leaf (500 mg/kg/day)	89.0^**^	1.0
Glibenclamide (0.175 g/kg/day)	87.1^**^	1.5

**Table 2:** Effect of insulin plant leaves and glibenclamide on postprandial blood glucose levels in dexamethasone-induced hyperglycemia

Groups	Mean	Std. deviation
Control	90.1	1.1
Dexamethasone (10 mg/kg/day)	182.8^***^	1.7
Insulin plant leaf (100 mg/kg/day)	170.0	1.4
Insulin plant (250 mg/kg/day)	127.1^**^	1.7
Insulin plant leaf (500 mg/kg/day)	120.8^**^	1.3
Glibenclamide (0.175 g/kg/day)	125.1^**^	1.6

## DISCUSSION

Diabetes mellitus induced by glucocorticoids is similar to type 2 diabetes mellitus, where insulin resistance constitutes an essential component. Further it may be mentioned that glucocorticoids also cause obesity, hypertension, hyperurecemia, increased plasminogen activator inhibitor – 1, low HDL (high-density lipoprotein) cholesterol along with glucose intolerance. The cluster of these abnormalities was coined as "metabolic syndrome" by WHO in 1999.[3]

Dexamethasone is a long-acting glucocorticoid with t½ more than 36 hours. According to a study, transgenic mice producing excess of 11–beta HSD develop typical features of the metabolic syndrome, suggesting that excess of cortisol in tissues might be responsible for the insulin resistance, a core feature of type 2 diabetes mellitus. Dexamethasone produces dose-dependent inhibition of insulin release caused by glucose, tolbutamide and other insulin releasers. [4] Therefore, only insulin sensitizers and insulin are likely to be effective in dexamethasone-induced hyperglycemia. Insulin sensitizers like Rosiglitazone and metformin are being evaluated for primary prevention of type 2 diabetes mellitus in high-risk patients.[5]

Plant products have been used in folk medicine and traditional healing systems and are being evaluated for their hypoglycemic effects. The study was planned to evaluate the insulin-sensitizing effects of the insulin plant (*Costus igeus*) in dexamethasone-induced hyperglycemia in male albino rats. In the present study, there was increase in the fasting and postprandial glucose level with 10 mg/kg/day of dexamethasone when compared to normal controls (*P*< .001, Kruskal-Wallis test). Reduction in the fasting and postprandial blood sugar levels with leaves of insulin plant in dexamethasone-induced hyperglycemia was comparable with that obtained with Glibenclamide 500 µg/kg at 250 mg/kg and 500 mg/kg of powdered leaves of the insulin plant (*Costus igeus*) (*P*< .01) (Mann-Whitney *U* test) [Figure 2].

The hypoglycemic action can be due to release of insulin, insulin-sensitizing action or a combination of both. Hence further studies need to be undertaken to determine the mechanism of action by measurement of either insulin or 'C' peptide level.

## CONCLUSION

The leaves of insulin plant (*Costus igeus*) reduced the fasting and postprandial blood sugar levels, bringing them down towards normal, in dexamethasone-induced hyperglycemia in rats. Reduction in the fasting and the postprandial blood sugar levels with leaves of insulin plant was comparable with that obtained with Glibenclamide 500 µg/kg at 250 mg/kg/day and 500 mg/kg/day of powdered leaves of the insulin plant (*Costus igeus*).
